# Design of a square-horn hybrid plasmonic nano-antenna array using a flat lens for optical wireless applications with beam-steering capabilities

**DOI:** 10.1038/s41598-024-75834-y

**Published:** 2024-11-07

**Authors:** Fatma E. Helmy, Ibrahim I. Ibrahim, Amany M. Saleh

**Affiliations:** Electronics and Communications Department, Faculty of Engineering, Helwan University, Cairo, 11795 Egypt

**Keywords:** Hybrid plasmonic optical nano-antenna, Optical phased arrays, Dielectric flat lens nano-antenna, Horn nano-antenna, Optical beam-steering device, Optical wireless applications, Engineering, Nanoscience and technology, Optics and photonics

## Abstract

This paper introduces a Hybrid Plasmonic Nano-Antenna (HPNA) with a gradient-index dielectric flat lens modeled with different materials to enhance and steer the radiation in a particular direction based on a phase shift array. Firstly, the design of hybrid plasmonic Nano-Antenna (NA) is introduced and analyzed considering different horn-shapes such as diamond, hexagonal, circular, rectangular, and square shapes. The commercial software Computer Simulation Technology-Microwave Studio (CST-MWS) is used to analyze the radiation characteristics of the plasmonic NAs at the standard telecommunication wavelength of 1,550 nm. The produced horn-shaped nano-antenna made up from gold cladding with low- and high-index dielectric materials of SiO2 and InGaAs, respectively. The gain of the Square Horn shape Hybrid Plasmonic Nano-Antenna (SHHPNA) achieves the greatest gain with a value of 10.7 dBi at the desired frequency and the return loss reached -18.09 dB due to the wide aperture area for SHHPNA, which results in a narrower beam-width and higher gain. Moreover, by using two different shapes of dielectric flat lens to enhance the antenna’s performance by improving directivity while correspondingly reducing beam-width, the gain is enhanced and reaches 16.7 for SHHPNA with a circular lens and 16.9 for SHHPNA with a rectangular lens compared with the traditional NA that equal to 9.03 dBi. The main lobe for SHHPNA with each lens is more directed, with Side Lobe Level (SLL) and Half Power Beam-Width (HPBW) of -13.1 dB and 16.5° for SHHPNA with a circular lens and -15.1 dB and 15.4° for SHHPNA with a rectangular lens, respectively. In addition, the array configuration was investigated, and the gain was found to be 21 dBi for the single row array of 4×1 and 23.2 dB for the array of 3×3. Moreover, the array of 4×1 and 3×3 with +90° showed gains of 18.6 dBi and 20.7 dBi, respectively, compared to traditional paper with gains of 11.20 dBi and 13.1 dBi.

## Introduction

For many applications, including free-space optical communications, Light Detection And Ranging (LiDAR), imaging, biological sensing, and special beam creation^1–3^, the optical beam-steering device is a crucial optical element. Because of this, Optical Phased Arrays (OPAs) have garnered a lot of attention lately^4–6^. Large-scale OPAs with rapid manipulation and adaptive steering provide a low-cost chip-scale technology for a variety of high-performance uses. Each element in a phased array antenna has a changeable phase. By setting the phase of each antenna element to a specific value, the signals can be merged constructively in one direction and destructively in the other. This allows for the modification of broadcast direction and antenna strength. Recently, several exciting scalable phased array structures and concepts have been developed for millimeter-wave bands^7^. Numerous researchers have investigated the principles, beamforming, steering, components, and configurations of OPAs for space optical communication in the optical range. In general, optical switches and phase controllers can provide two-dimensional (2-D) beam-steering at a given wavelength, whereas beam-steering is accessed by actively switching among several Waveguide Antennas (WGAs) with varied diffraction angles^8^. A phase-shifter with a wide phase-shifting range, high phase-shifting efficiency, small footprint, and low optical loss should be developed in order to realize large-scale photonic integrated OPAs. In this situation, the lens focuses the incident light to a target point, thereby acting as a passive phase-shifter. This lens exhibits focused signal power directed to a particular sub-region of the antenna array, and concentrated signal power directed to the front end, resulting in high gain and directivity when coupled with antennas. The lens is an effective tool for using optical beamforming systems because of these features. Different kinds of metamaterial (gradient-index) lenses have been created in^9–16^. In^9^, Fresnel zone plate lenses are offered to rectify the feed antenna’s phase, resulting in a concept that is naturally narrowband at specific locations. Luneburg lenses are introduced in^10,11^ as spherical or hemispherical gradient-index lenses for which only one beam scanning plane is authorized. Alibakhshikenari et al.^12^ presented a survey that provides an overview of techniques and technologies, including metamaterial, metasurface, and substrate integrated waveguides. The presented antenna designs are implemented on different substrate layers, including Polyimide, Silicon, Graphene, and gallium arsenide, to enable integration on integrated circuits. Several of the provided antennas use innovative excitation techniques. To prevent surface wave propagation and lower substrate loss, for instance, an open-circuited microstrip line that is electromagnetically connected to radiating elements through thin dielectric slots is used. Other methods, like as substrate integrated waveguides, have been demonstrated to significantly reduce loss and attenuate surface waves. Alibakhshikenari et al.^13^ concentrated on artificial metamaterial transmission lines for the realization of various cost-effective, simple-to-design and manufacture, and mass-produced antenna structures with miniaturized dimensions, wide bandwidth, high radiation gain and efficiency, wide range of scanning ability, and low profile, as well as some of their most common and relevant applications. A planar dielectric lens is constructed in^14^, and the performance of the lens is simulated using HFSS. The performances of a plane layer dielectric lens antenna are evaluated when a microstrip patch antenna is employed as the feeding antenna. The distance between the lens and the feeding antenna is discussed. The authors investigate the deviation from the central axis as well as the non-parallel between the feeding antenna and the lens towards the lens antenna. A plane dielectric lens antenna with beam-steering is feasible. To maintain a flat antenna profile that is much thinner than existing traditionally shaped lenses while achieving beam-scanning in both planes and enabling the broadband process, a design based on a switched-beam array antenna concept with an inhomogeneous dielectric flat lens proposed with different materials was presented in^15^. In^16^, thorough laboratory testing of novel dielectric flat lens antennas for high data throughput 5G wireless communication systems operating in the 60 GHz band is provided together with Low-Temperature Co-fired Ceramics (LTCC) production. As a result, inhomogeneous gradient-index dielectric flat lenses in the optical range for wireless optical communication were designed and numerically simulated. The greatest feasible gain, beam-scanning capability, bandwidth efficiency, and overall performance of this lens. Ndao et al.^17^ introduces flat metalenses, with each subwavelength-scale meta-atom on the metasurface acting as an antenna or a waveguide for a phase shift. Because the elements on a metalens are at varied distances from the central focus, a separate phase delay is required.

To distinguish between active and passive materials, particularly in the context of lenses: Firstly, active materials defined by materials can modify properties including electrical conductivity, light transmission, and mechanical deformation in response to external stimuli (e.g., electric field, light, temperature). For example: 1. Liquid crystal lenses are frequently utilized in active eyewear, including 3D glasses for movies and televisions. When an electric field is applied, these lenses’ optical characteristics (such as transparency and refractive index) change. 2. Electrochromic lenses when a voltage is applied, these lenses alter in color or opacity. They have applications in rearview mirrors, smart windows, and eyewear. 3. Active infrared sensors that use pyroelectric sensors to measure heat energy. The sensor initiates an action (such as turning on lights or an alarm) when the temperature differential between the sensors changes (for example, as a result of movement). Secondly, passive materials exhibit specific features, either statically or naturally, but do not actively respond to external stimuli. For example: 1. Passive optical materials called polarized lenses are utilized in sunglasses. They minimize glare from surfaces such as water or highways by blocking particular orientations of light wavelengths, which are often horizontal. They don’t dynamically alter their characteristics. 2. Glass lenses: Conventional glass lenses possess fixed optical properties, such as dispersion and refractive index, and are passive. They are not conditionally adaptive. 3. Passive infrared (PIR) sensors, in contrast to active infrared sensors, do not emit infrared radiation. They identify the infrared radiation that warm objects (including humans) in their area of vision are currently emitting. In conclusion, active materials in lenses can dynamically differ in their optical properties, while passive materials maintain fixed characteristics.

An 8×8 hybrid plasmonic NA array shows beam-steering techniques at 1550 nm in^18^. By employing a Deep Neural Network (DNN) with or without a lens to forecast the correct feeding phases of the 64 elements, the phased array antenna guides the beam. Ghaffari et al.^19^ uses an integrated optical system that combines a reflective meta-lens with five switchable NAs to provide optical beam-steering at the standard telecommunication wavelength of 1550 nm. A Rotman lens was used in^20^ to create switchable phase-shifting; by using a non-absorptive power splitter and increasing the number of input ports on the Rotman lens, respectively, the realized gain and switchable beam count may be enhanced. Zhang et al.^21^ takes into account pattern multiplication while assessing the antenna array’s overall field pattern. The mutual interaction between antenna elements has been taken into consideration in this work. Optical NA arrays are described in terms of transmission, reception, and reflection in a recent study by Sangwan et al.^22^. The fundamental components that affect how single optical antennas are built are then specified and modeled. The mutual interaction of the optical NA is studied to guide the design of small antenna arrays. Finite element methods are used to objectively assess the performance of various beamforming strategies. Additionally^23^, uses individually regulated OPAs to phase calibrate manufacturing flaws, thermal, cross talk, and electrical drifts.

Scientists have become more interested in Surface Plasmon Polaritons (SPPs) over the years since they provided a new, promising approach for the next generation of nanotechnologies. At the metal-dielectric layer interface in the visible and far-infrared (IR) spectrum, Plasmonic Waveguides (PWs) and waveguide-fed nano-antennas support highly localized optical fields^24,25^. However, due to the metallic attenuation, PWs’ light propagation length is much shorter than that of optical fibers and Dielectric Waveguides (DWs)^26–29^. The benefits and drawbacks of PWs and DWs are basically complementary in several ways^30^. While DWs are lossless in one sense, the size of their mode is constrained by the diffraction limit. PWs can concentrate light up to the diffraction limit but suffer significant propagation losses. In contrast to PWs, Hybrid Plasmonic Waveguides (HPWs) have been created and demonstrated to offer a superior trade-off between the inevitable metallic loss and fundamental mode confinement in a nano-scale layer. The Surface Plasmon (SP) mode provided by the PW couples with the TM mode of the DW in the midpoint of the low refractive index layer (coupling region) when two waveguides (PW and DW) are brought close to one another. Fundamentally, it has been demonstrated in earlier research that HPWs can sustain a hybrid plasmonic quasi-transverse magnetic mode^31–36^, which is constrained in a layer of low index dielectric sandwiched between the noble metal and layer of high index dielectric since the SP mode is TM in nature. Thus, by introducing the HPWs, the benefits of both DWs (lossless characteristic) and PWs (resolving the scattering limit) have been merged. Additionally, two even and odd linked modes for similar PW and DW make up the propagating hybrid mode. A Circular Hybrid Plasmonic Waveguide-Fed Nano-Antenna (CHPWFNA) for use at the typical telecommunication wavelength of 1,550 nm using numerical methods has been introduced and 9.03 dB of gain was attained in^37^. Additionally, the array configuration has been examined, and the realized gain has been determined by 11.20 dB for the single row array of 4×1 and 13.1 dB for the array of 3×3 with Δ ϕ = +90◦ under consideration.

In this paper, an optimal hybrid plasmonic nano-antenna with a flat lens with beam-steering capability for optical wireless applications was introduced at the standard telecommunication wavelength of 1550 nm (193.5 THz). In section 2, the design considerations and simulation methodology of the plasmonic NAs with various horn geometries and the dielectric flat gradient-index lens are presented with a comparison with the previously published results. The results are presented and discussed in Section 3. Finally, Section 4 summarizes the findings.

## Design considerations and simulation methodology

**Fig. 1:**
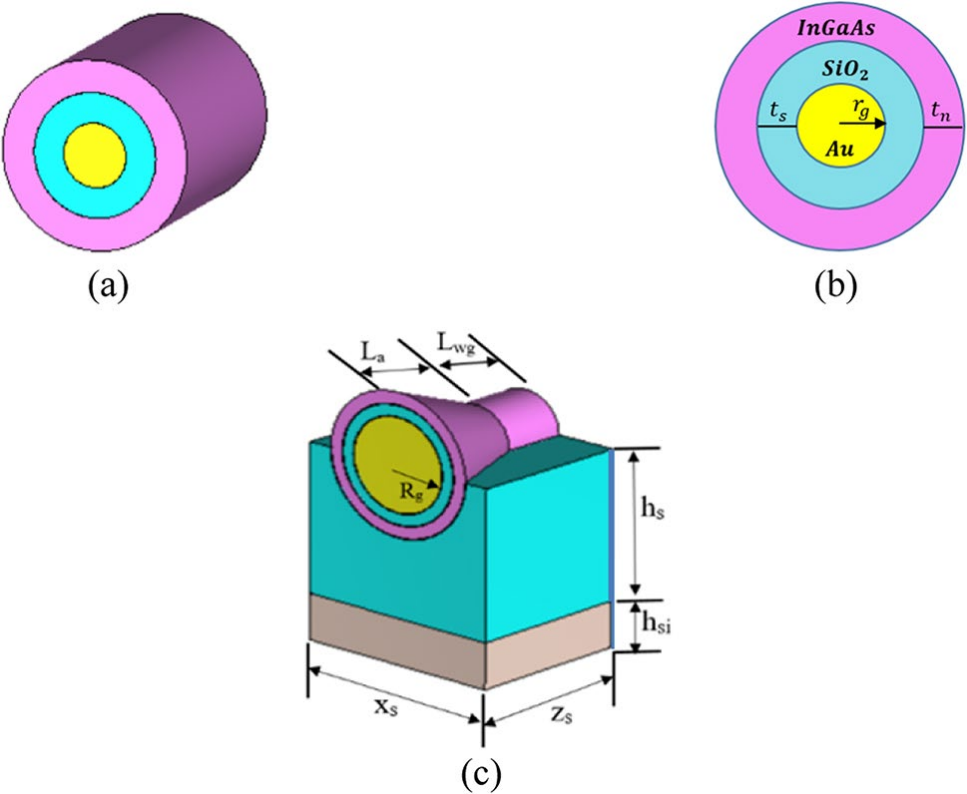
(**a**) 3D schematic view of the circular hybrid plasmonic wave-guide (CHPW), (**b**) cross-section view of CHPW, (**c**) 3D schematic illustration of the proposed circular hybrid plasmonic nano-antenna (CHPWFNA)^37^.

As illustrated in Fig. 1c, a 3D schematic illustration of the introduced CHPWFNA in^37^, which is excited by the Circular Hybrid Plasmonic Waveguide (CHPW) shown in Fig. 1a, b. Analytical derivation has been made of the dispersion relation of the fundamental TM_01_ mode of the CHPW^37^. CHPW is made up of a metal wire layer of gold with low- and high-index dielectric materials of SiO_2_ and InGaAs, respectively. A decreased cladding fiber is encircled by a thin coating of gold in this design, which is an improved structure for a bare metal wire waveguide with a hybrid mode. According to the hybrid plasmonic waveguide theory, excited SPPs are concentrated in low-index layers, and their amplitudes exponentially decline in high-index metal and dielectric layers. Additionally, the low refractive index layer’s precise port mode (TM_01_ mode) is determined using the eigenmode solver. To describe the CHPW’s characteristics, the propagation length L_P_, the normalized mode A_mod_, and the figure of merit (FoM) defined by^37^:

1 $${L}_{P}=\frac{{\lambda }_{o}}{4\pi \left|{k}_{eff}\right|}$$

2 $${A}_{mod}=\frac{{A}_{eff}}{{\lambda }_{0}^{2}}$$

3 $$FOM=\frac{{L}_{P}}{\sqrt{{A}_{eff}/\pi }}$$

where λ_0_ is the free space wavelength, k_eff_ is the imaginary part of the effective refractive index, and A_eff_ is the effective mode area. The propagation length (L_P_) is determined by the value of the imaginary part of the effective refractive index, which in turn depends on the characteristics of the Au layer, including its permittivity and thickness. A_mod_ and FoM were obtained, with values of 0.05 and 41.02, respectively. A successful CHPW design must simultaneously have a small mode area and a long propagation length, which will need a trade-off. Based on the results, it is evident that the suggested waveguide may successfully break the inescapable loss-confinement trade-off, which is desirable for integrated circuits, with such a small mode area and long propagation length of SPPs. The gold nanowire’s radius is r_g_=80 nm. The SiO2 and InGaAs layers have thicknesses of t_s_ = 75 nm and t_n_ = 80 nm, respectively, and relative permittivities of ε_s_ = 2.1055 and ε_n_ = 13.84. The relative permittivity (g) of the Au layer is calculated using Johnson-Christy data^38^. The CHPW has a length of L_wg_=375 nm. The horn antenna, coupled with a bigger hole, is extremely effective in the RF area because it can create a consistent phase front to improve directivity and gain^39^. The produced horn-shaped nano-antenna has a larger radius of the gold with R_g_ = 300 nm. The SiO_2_ and InGaAs layers in the horn shape have thicknesses of t_s_ = 75 nm and t_n_ = 80 nm, which correspond to their thicknesses in CHPW. The lengths of the horn shape L_a_ and SiO_2_ layer h_s_ are 575 nm and 830 nm, respectively. A silicon wafer is a thin flat slice of massive, single, and defect-free crystalline silicon that serves as a suitable substrate platform. Silicon (Si) is typically an excellent choice because silicon-on-insulator (SOI) wafers are commonly employed in silicon nanophotonics. The thickness of the Si substrate is h_Si_ = 220 nm. The proposed nano-antenna has dimensions of x_s_ × z_s_ = 1200 × 950 nm^2^. The radiation properties of the plasmonic NA at the frequency of 193.5 THz (λ= 1550 nm) are investigated using the commercial software of Computer Simulation Technology-Microwave Studio (CST-MWS)^40^.

### The hybrid plasmonic nano-antennas geometrical design

**Fig. 2:**
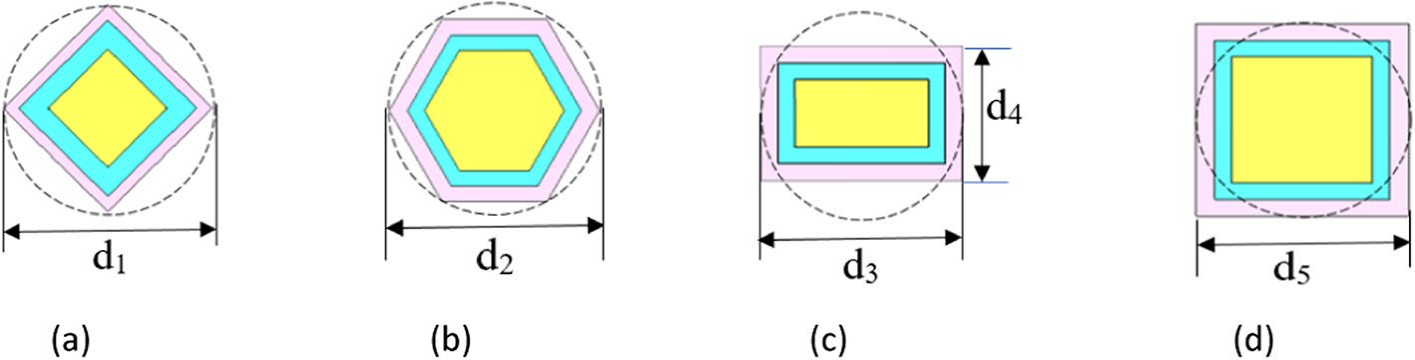
The geometrical structures of various horn forms (**a**) diamond, (**b**) hexagon, (**c**) rectangle, (**d**) square.

In this work, some parameters for the CHPWFNA are optimized using particle swarm optimization (PSO) to achieve the maximum directivity and gain of the proposed nano-antenna^41^. The optimized parameters are the lengths of the SiO_2_ layer (h_s_) and the Si substrate (h_Si_), which are equal to 970.78 nm and 299.81 nm, respectively. This section’s objective is to test several horn shapes, including diamond, hexagon, rectangle, optimized circle, and square, to see how they affect NA characteristics. Figure 2 depicts the geometrical structures formed by different horn shapes. The dimensions of the diamond, hexagon, rectangle, and square horn forms of (d_1_, d_2_, (d_3_, d_4_), and d_5_) are equal to (600, 600, (600, 300), and 600) nm, respectively. The radiation properties of plasmonic NAs produced with CST-MWS were compared to the circle horn shape previously published in^37^. To investigate the performance of the developed NAs, the gain should be calculated. The term gain describes how much power is transmitted in the direction of peak radiation to that of an isotropic source. The gain of an antenna is defined as the ratio of power delivered to the antenna and the power that is radiated and can be calculated by^42^:

4 $$G = \frac{{U\left( {\theta ,\emptyset } \right)}}{{P_{in} /4\pi }}$$

where U(θ,φ) is the radiation intensity, and P_in_ is the electrical power received by the antenna from the transmitter.

### Dielectric flat gradient-index lens design

The radiation is enhanced and directed in a certain direction using the simulated inhomogeneous dielectric flat lens made of various materials. Dielectric multi-material lenses provide several advantages over metalenses or metasurfaces:

- *Material properties* Dielectric Multimaterial lenses are manufactured from dielectric materials that have minimal optical losses. Dielectrics have a wide transparency range and can be designed to have certain refractive indices. Metalenses, also known as metasurfaces, are a specific type of metasurface-based flat lens. Metalenses are subwavelength nanostructures made from metal. Metals suffer from high optical losses, particularly in the visible and near-infrared regions.
- *Simplicity of fabrication* Dielectric Multimaterial Lenses can be manufactured using regular lithography and nanoimprinting procedures, making them simpler than sophisticated 3D metamaterials. Metalenses and metasurfaces are difficult to fabricate due to the complicated nanofabrication techniques involved.
- *Integration and tunability* Dielectric Multimaterial Lenses can be readily modified by adjusting the refractive index or dielectric layer thickness. Metalenses and Metasurfaces are difficult to tune and typically have a set wavelength of operation.
- *Correction of aberrations* Dielectric Multimaterial Lenses: Their broadband performance allows for more effective chromatic aberration correction. Metalenses and metasurfaces have restricted spectral bandwidth, which causes chromatic aberrations.
- *Applications* Dielectric Multimaterial Lenses include imaging, holography, and wavefront control. Metalenses and metasurfaces have limits in image quality but excel in other areas such as compactness, varifocal properties, and extended depth of field.

**Fig. 3:**
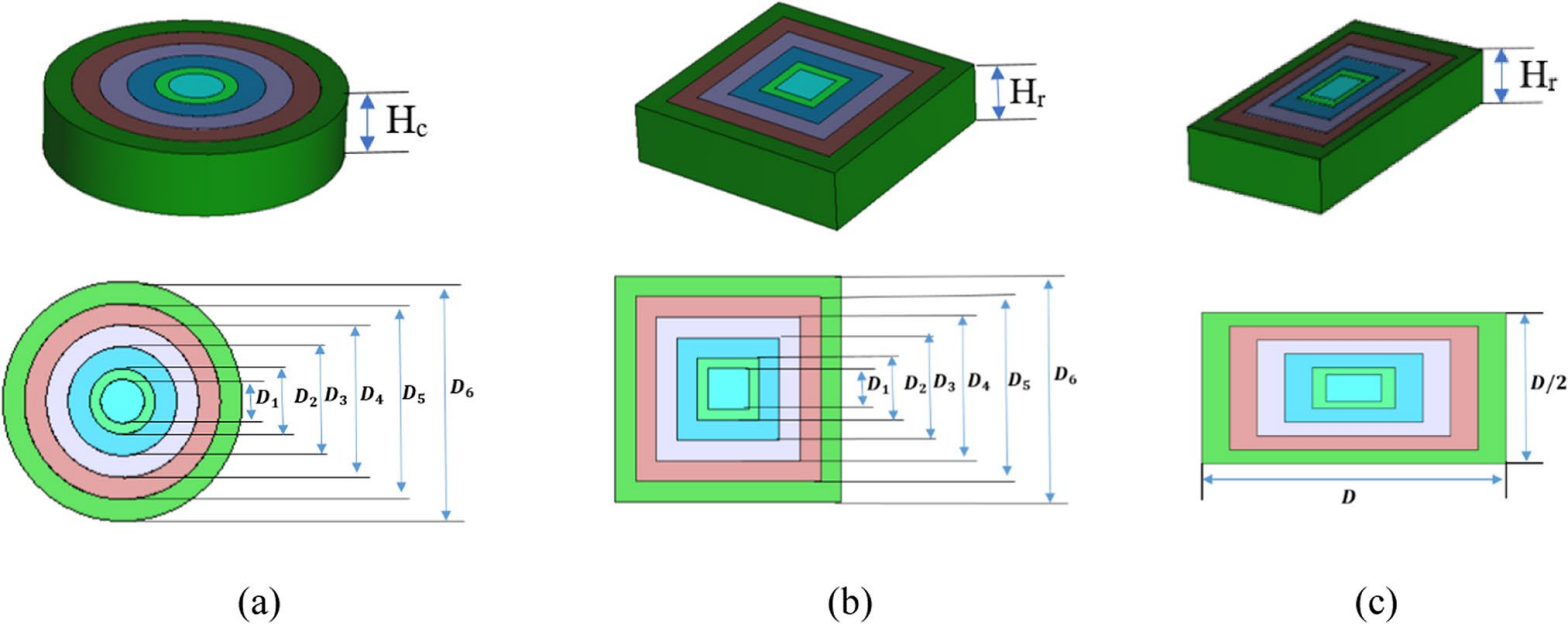
Dielectric flat gradient-index lens structure. (**a**) circular lens, (**b**) square lens, (**c**) rectangular lens.

As a result, dielectric multimaterial lenses are preferable than metalenses and metasurfaces in terms of performance, ease of manufacture, and range of applications^43–53^. However, the decision is based on the specific requirements and trade-offs for a certain application. The theoretical design of the dielectric flat lens and its operating concept are covered in^9^. Six concentric rings of various permittivity materials (r) make up the theoretical lens design, which is intended to provide the necessary phase delays for improving the radiation pattern throughout the lens when lighted from the focal position. As a result, the materials’ permittivity in the introduced circle lens’ adjacent rings were as follows: ${\varepsilon }_{{r}_{1}}$ > ${\varepsilon }_{{r}_{2}}$ > ${\varepsilon }_{{r}_{3}}$ > ${\varepsilon }_{{r}_{4}}$> ${\varepsilon }_{{r}_{5}}$ > ${\varepsilon }_{{r}_{6}}$ where the maximum permittivity ${\varepsilon }_{{r}_{1}}$ at the center of the lens and a steady decline to the minimum permittivity ${\varepsilon }_{{r}_{6}}$ at the outer ring. Lens design and manufacture with uniform thickness for a flat form provided by Eqs. 5 and 6. The radii (R_i_) for each dielectric zone and the thickness of the lens H that is proportional to the two adjacent permittivities at the lens can be found by^9,10,15,16,54,55^:

5 $${R}_{i}=\sqrt{2Fi\left(\frac{\uplambda }{P}\right)+{\left(i\frac{\uplambda }{P}\right)}^{2}} , i=2, 3, \dots .., P$$

6 $$H=\frac{\uplambda }{P(\sqrt{{\varepsilon }_{ri}}-\sqrt{{\varepsilon }_{ri-1}}} , i=2, 3, \dots .., P$$

where λ is the design wavelength, P is the phase correcting index and is equal to 6, and F is the focal length. As illustrated in Fig. 3a, a Rogers TMM6 dielectric substrate^16^ was used to create the required permittivity profile, with a maximum permittivity value of 7.1 at the center of the lens and a smooth, continuous decline to 2.9 at the edges. The various dielectric flat lenses have characteristic features such as 7.1, 6.79, 6.01, 4.99, 3.92, and 2.9. The focal length F and thickness of the produced lens H_c_ with values of 1937.5 nm (D/4) and 2170 nm (1.4λ), correspondingly. The produced dielectric flat gradient-index lens has the outermost diameter of lens D_6_ = D = 7750 nm (5λ)$.$ As shown in Fig. 3a, (D_1_, D_2_, D_3_, D_4_, D_5_, D_6_), which are equal to the numbers (1409, 2113, 3522, 4931, 6340, and 7750, respectively), determine the outer and inner diameters of the rings of the circular lens^55^. Regarding the circular lens, a square lens is created with dimensions equal to the diameters of the lens as shown in Fig. 3b. Moreover, the thickness of the introduced square lens H_r_ is equal to the H_c_ of the circular lens. Furthermore, a rectangular lens forms with a width equal to the square side length and a length equal to half of that value, as illustrated in Fig. 3c.

### Array of the squared horn shape nano-antenna

**Fig. 4:**
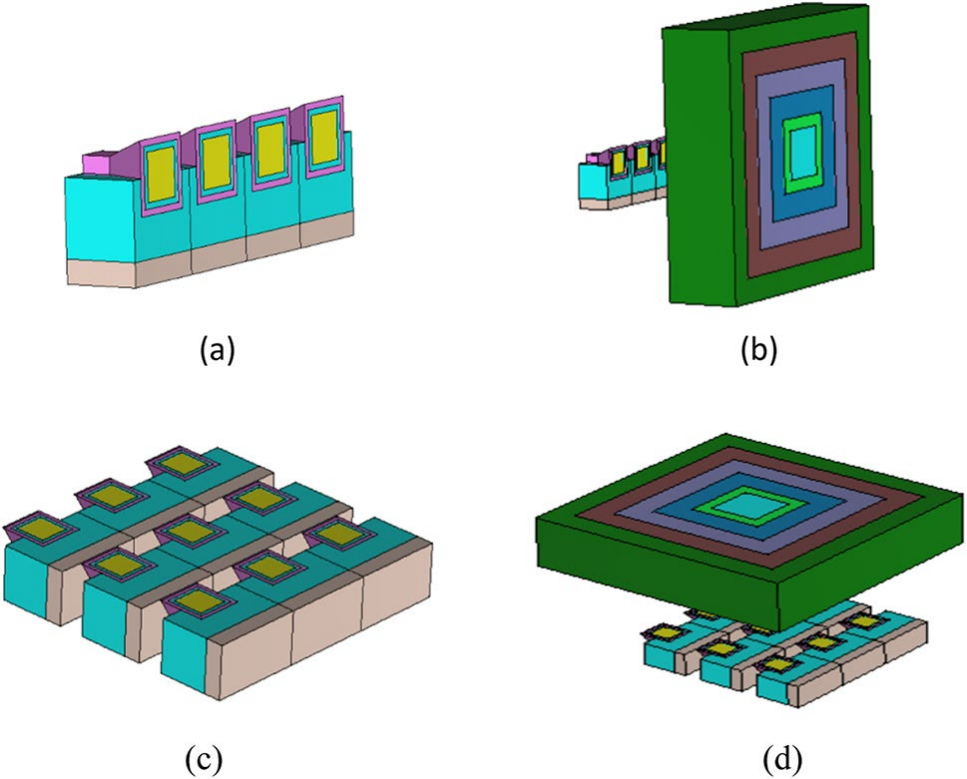
3D schematic configuration of the introduced arrays of SHHPNA (**a**) 4×1 array NA without the lens, (**b**) 4×1 array NA with the square lens, (**c**) 3×3 array NA without the lens, (**d**) 3×3 array NA with the square lens,

Beam-steering capabilities, which allow for dynamic control of nano-antenna radiation patterns, can benefit applications such as optical communication, holography, imaging, sensing, and LiDAR^56–61^. The topic of beam-steering using a nano-antenna array is remarkable. It involves controlling a beam of light using an array of nano-antennas designed to produce an antenna with a high gain that is also perfect for energy harvesting. The beam can be directed in a certain direction by adjusting the phase and amplitude of the light waves generated by each nano-antenna. By increasing the number of the introduced square horn-shaped nano-antenna elements, the effectiveness of the suggested nano-antenna for energy harvesting applications is confirmed. Fig. 4a and c show 3D schematic configurations of the 4 × 1 and 3 × 3 arrays of square horn-shaped hybrid plasmonic nano-antenna (SHHPNA) which give the highest results compared to the other horn shapes. The spacing between the adjacent nano-antennas is also an essential consideration. The spacing between two consecutive elements is predetermined to be λ_eff_ > 420 nm, where λ_eff_ > (λc / 2 n_eff_), λ_c_ denotes the bandwidth’s central wavelength, and n_eff_ is the real part of the refractive index of the surrounding material. However, the distance between two neighboring nano-antennas is 600 nm > 420 nm, which is sufficient to overcome the coupling effect between them because the antenna footprint is 1200 nm along the x-axis.

In this paper, we present an OPA design with a dielectric flat gradient-index lens in which only a subset of OPA antenna elements are used for 2-D beam-steering, significantly reducing overall OPA energy consumption and eliminating the requirement for electrical wiring within the aperture. The introduced OPA designs formed from the 4×1 and 3 × 3 arrays of the SHHPNA with a dielectric flat gradient-index lens modelled with different materials to steer and enhance the radiation in a particular direction as depicted in Fig. 4b and d, respectively. Beam-steering can be accomplished by adjusting the relative phase of the antenna elements, the lens’s varied permittivity values provide a phase with a linear slope that steers the beam correspondingly.

## Simulated results and discussion

This section discusses and evaluates the performance of the previously introduced hybrid plasmonic NAs with various horn forms. The radiation patterns typical of the square horn-shaped NA with the circular, square, and rectangular dielectric flat lenses are then discussed. Finally, beam-steering outcomes using a square horn-shaped NA array will be presented.

### The hybrid plasmonic nano-antenna with different horn-shapes

**Fig. 5:**
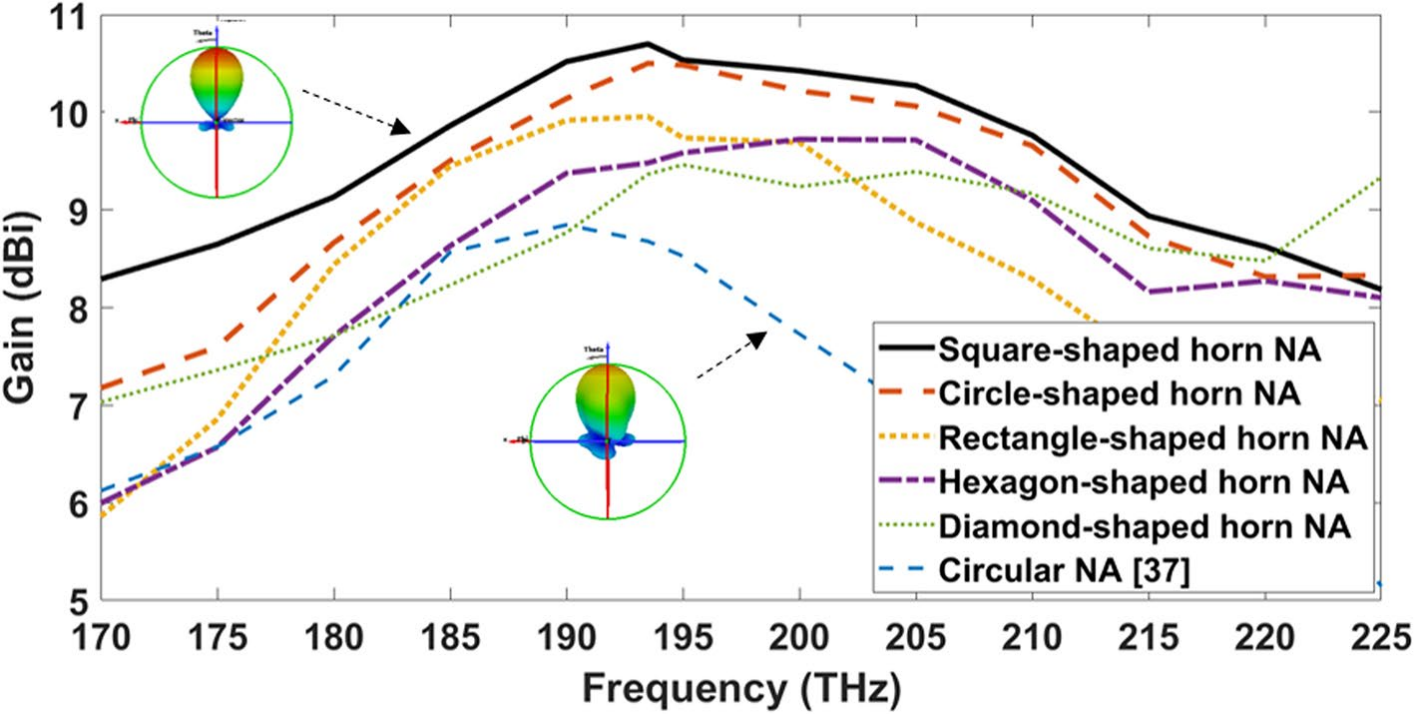
Gain over the frequency range of different hybrid plasmonic NAs

The geometrical specifications of the antenna are the primary determinants of its directivity. Figure 5 depicts the gain of the produced hybrid plasmonic NAs with different horn shapes over the frequency range of 170 to 225 THz. It is clear that the SHHPNA achieves the greatest gain with a value of 10.7 dBi at the desired frequency. The 3-D radiation patterns at the frequency of 193.5 THz of the introduced SHHPNA and the NA introduced in the published study^37^ are inserted inside Fig. 5. However, the main lobe of SHHPNA is smooth and radiates vertically without bidirectional radiation, with a Side Lobe Level (SLL) of -13.7 dB. Table. 1 displays the radiation pattern characteristic for the introduced nano-antennas with varied horn shapes to summarize the comparison between them at λ=1550 nm. The newly developed circle-shaped horn NA with certain optimized values has an increased gain of 1.47 dBi over the NA in the literature review^37^. Furthermore, the Half-Power Beam-Width (HPBW) of SHHPNA is found to be 52.2°, whereas the rectangle-shaped horn NA is 50.4°. Additionally, when compared to other structures, the reflection coefficients (S_11_) of SHHPNA equal -18.09, achieving the best matching at the frequency of 193.5 THz. Moreover, the hexagonal horn-shape NA attained a gain of 10 dBi, which is higher than the gain of diamond horn-shape NA with 9.83 dBi. This improvement is due to a change in the geometrical parameters, which allows for the usage of additional corners^62^. On the other hand, the radiation pattern characteristic is improved with the SHHPNA due to the homogenous distribution of the supported field intensity and wide aperture area for SHHPNA that cause the results in a narrower beam-width and higher gain. The most significant disadvantage of nanoantenna corners in diamond, rectangle, square, and hexagonal shapes is the complexity of their fabrication due to nanometer scale.

**Table 1:** The radiation characteristic comparison for various horn-shape NA at λ=1550 nm.

Horn shape	Square-shaped horn NA	Circle-shaped horn NA	Rectangle-shaped horn NA	Hexagon-shaped horn NA	Diamond-shaped horn NA	Circular NA^37^
					
Gain (dBi)	**10.7**	10.5	10.2	10	9.83	9.03
Radiation efficiency (%)	**97.6**	95	96.7	95.5	93	92.7
Return loss (dB)	**-18.09**	-17.6	-18.07	-17.03	-14.2	-12.6
SLL (dB)	**-13.7**	-10.9	-7.6	-8.1	-8.9	-9.7
HPBW (°)	52.2	51.8	**50.4**	55.1	57.7	53.9

Significant values are in [bold].

### Square horn-shape nano-antenna with different dielectric flat lenses

**Fig. 6:**
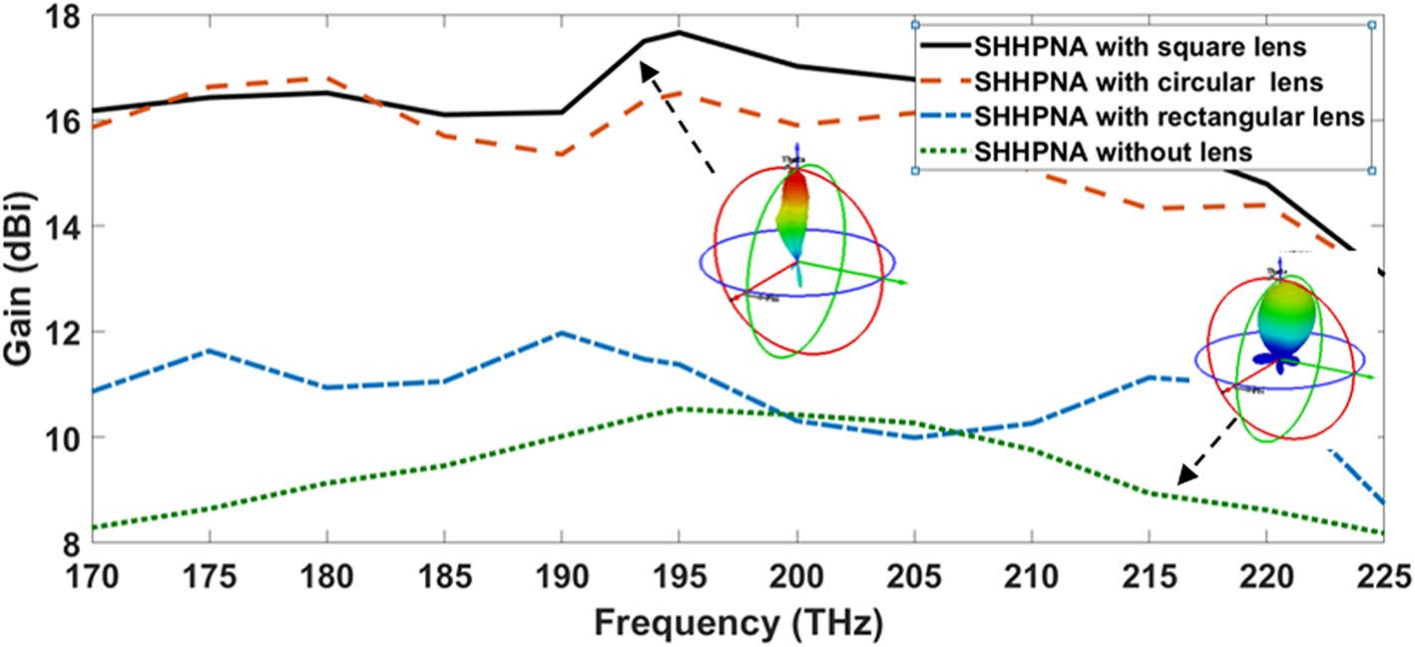
Gain of the introduced SHHPNA versus the frequency range

**Fig. 7:**
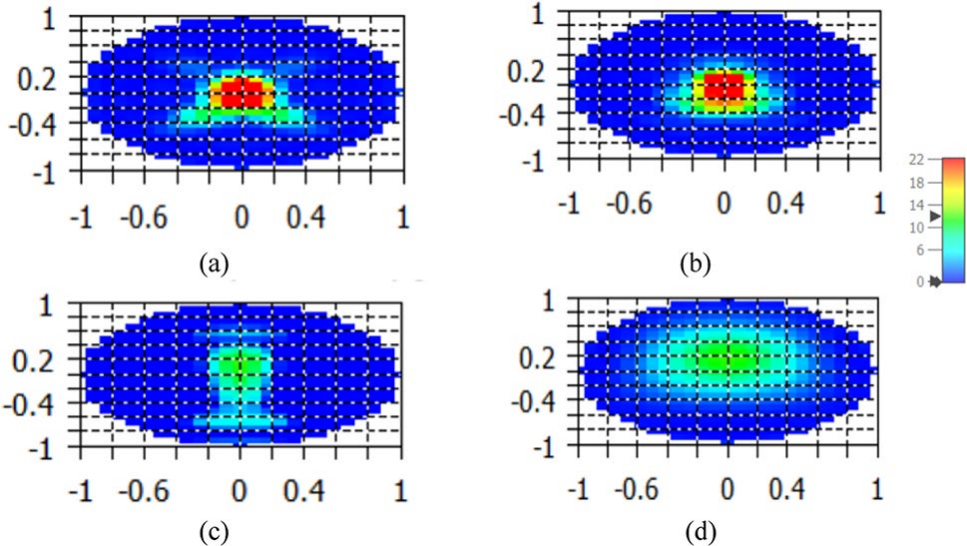
Far-field of the radiation pattern of the SHHPNA at 1550 nm (**a**) with the square lens, (**b**) with the circular lens, (**c**) with the rectangular lens, (**d**) without lens.

This section discusses the effect of different dielectric flat lenses on increasing NA directivity while simultaneously reducing beam-width while considering the SHHPNA. Figure 6 displays the gain of the examined SHHPNA with the square, circular, and rectangular lens over the frequency range in comparison to the SHHPNA without lens. The achieved gain of the dielectric flat lens NA with SHHPNA is increased to 12.5 dBi with the rectangular lens, 16.7 dBi with the circular lens, and 16.9 dBi with the square lens, as compared to 10.7 dBi for stand-alone SHHPNA without lens at 193.5 THz. The utilization of the low-loss substrates led to this improvement. The 3D radiation pattern of SHHPNA without the lens and with the square lens at 1550 nm are inserted inside Fig. 6 in the same field electromagnetic display scales for accurate comparison. Furthermore, the radiation pattern’s far-field at 1550 nm enhancement is observed in Fig. 7a of the SHHPNA with the square lens in contrast to Fig. 7b–d of the SHHPNA with the circle, rectangular lens, and without a lens, respectively.

**Fig. 8:**
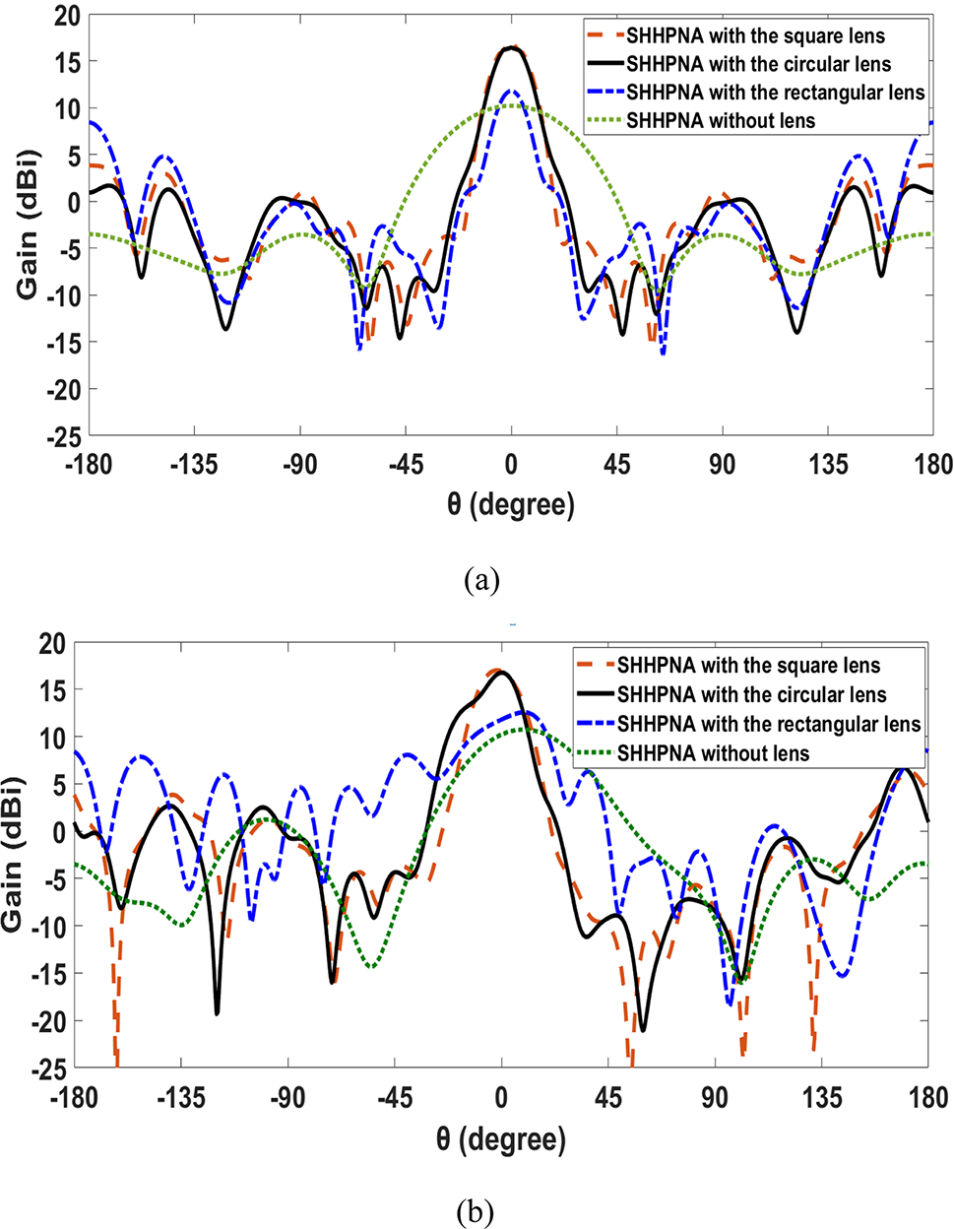
2-D radiation patterns of introduced SHHPNAs at 193.5 THz (**a**) in the x–z plane (φ = 0°), (**b**) in the y–z plane (φ = 90°),

Figure 8 depicts the 2-D radiation patterns at 193.5 THz of the previously introduced SHHPNA with and without lenses in the x–z plane (φ=0°) and y–z plane (φ=90°), respectively. The major lobe for SHHPNA with circular and square lenses are more directed, with SLL and HPBW of -13.1 dB and 16.5° for SHHPNA with circular lens and -15.1 dB and 15.4° for SHHPNA with square lens, respectively. However, some back radiation occurs as a result of lens reflections. In order to summarize, Table 2 shows the comparison between the introduced SHHPNA with and without lenses at λ=1550 nm. Moreover, the square lens outperforms the circular and rectangular lenses in terms of radiation pattern performance due to its greater aperture.

**Table 2:** The radiation performance for the SHHPNA with different shapes of lenses at λ=1550 nm.

SHHPNA	Gain (dBi)	SLL (dB)	HPBW (°)
With square lens	**16.9**	**-15.1**	**15.4**
With circular lens	16.7	-13.1	16.5
With rectangular lens	12.5	-3.4	14.1
Without lens	10.7	-13.7	52.2

Significant values are in [bold].

**Fig. 9:**
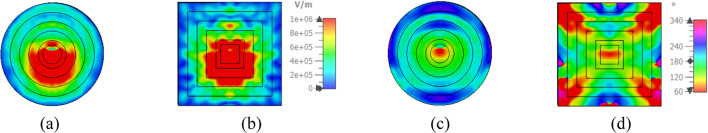
The amplitude and phase of the simulated electric field at 1550 nm for the SHHPNA apertures produced by: (**a**, **c**) the circular lens, (**b**, **d**) the square lens.

It can be observed that the square lens exhibits a little higher gain than the circular lens. Figure 9a, b depicts the amplitudes of the simulated electric field at 1550 nm for the SHHPNA apertures produced by square and circular lenses, respectively. Furthermore, the phases of the simulated electric field generated by the SHHPNA with the square and circular lenses, respectively, are depicted in Fig. 9c, d. They occur when viewed from the plane of the surface in front of the lens apertures. The results of the simulation demonstrate that the designed square lens performs better than the circular lenses in transforming the incident wave’s wave-front into a plane wave with less backscatter.

**Fig. 10:**
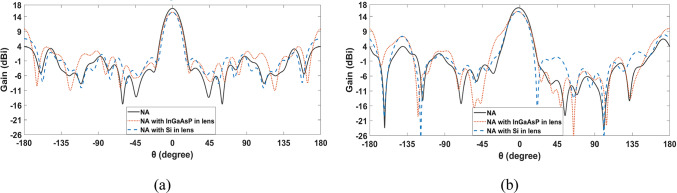
2-D radiation patterns at 193.5 THz of the SHHPNA with the previously introduced square lens and different materials in the lens: (**a**) in the x-z plane (φ=0°), (**b**) y-z plane (φ=90°)

Assuming that the dimensions are not optimized, the investigation of passive materials such as silicon (Si) instead of the inner material of the lens, which has a permittivity of 7.1, was initiated. Also, active materials like indium gallium arsenide phosphide (InGaAsP) were used instead of the lens’s outer material, which has a permittivity of just 2.9^63–65^. Figure 10 shows the 2-D radiation patterns at 193.5 THz of the SHHPNA with the previously introduced square lens, silicon (Si) inner material, and indium gallium arsenide phosphide (InGaAsP) outer material, respectively.

**Fig. 11:**
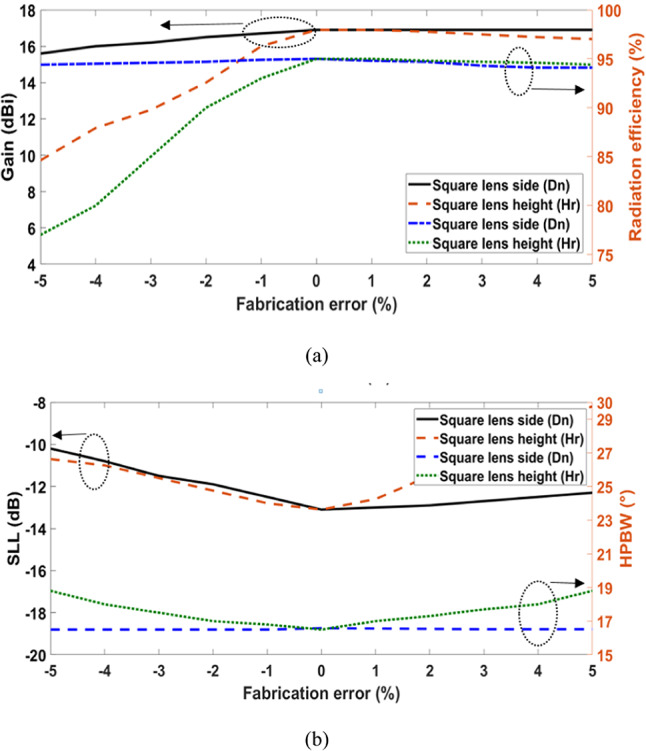
Variation of the fabrication error versus (**a**) gain and radiation efficiency, (**b**) SLL and HPBW.

Confirming the feasibility of producing the proposed nano-antenna requires research on the deviation tolerance and fabrication technique. Even with the remarkable technological advances in fabrication methods, it is not feasible to build a building with precisely the right proportions as intended. Consequently, to take into consideration the deficiencies in the fabrication, the far-field characteristics of the nano-antenna have been calculated for up to at least ±5% of dimension variations. The fabrication error variation is plotted against gain and radiation efficiency in Fig. 11a, while SLL and HPBW are shown in Fig. 11b.

**Fig. 12:**
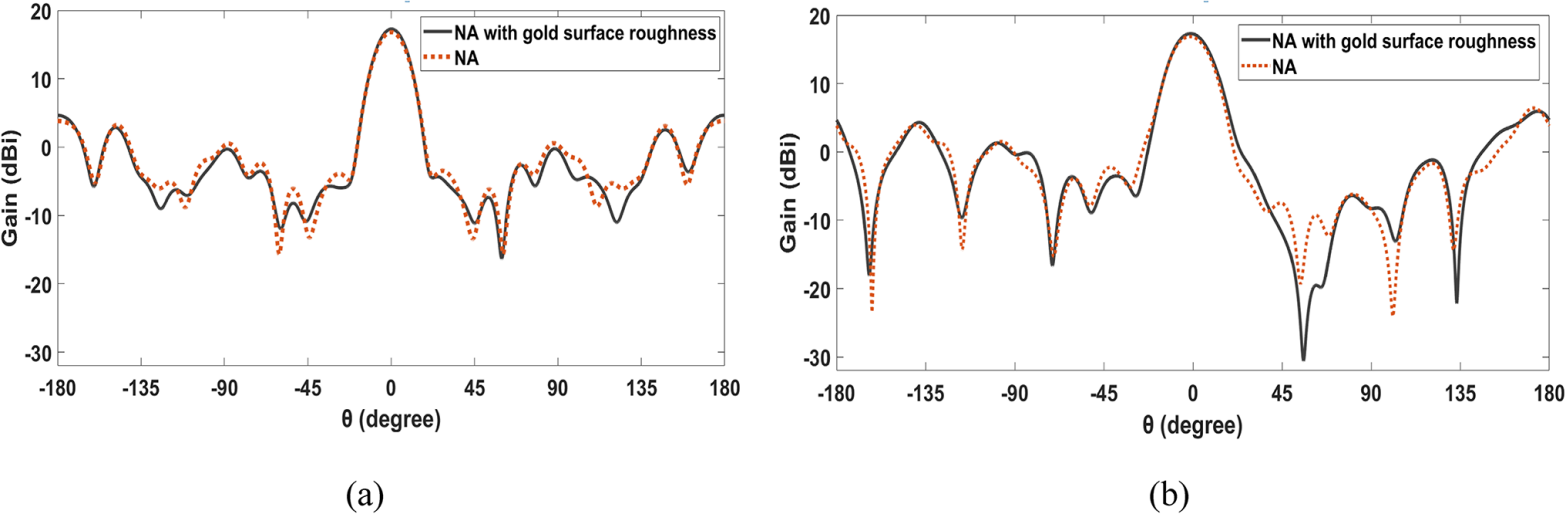
Effect of gold surface roughness on the introduced SHHPNA with the square lens at 193.5 THz (**a**) in the x–z plane (φ = 0°), (**b**) in the y–z plane (φ = 90°),

Surface roughness is a critical factor in the performance of optical systems, especially lenses. A recent study has examined the impact of gold surface roughness on lens performance during fabrication. The findings highlight that nanometer-scale surface roughness significantly influences the performance of the proposed NA with a square lens. Specifically, a gain of 17.3 dBi was achieved using a gold layer with a random surface roughness fluctuation of 0.5 nm on the lens. Figure 12a depicts the effect of gold surface roughness on the SHHPNA with a square lens at 193.5 THz in the x–z plane (φ = 0°), while Fig. 12b shows the same effect in the y–z plane (φ = 90°). Additionally, the study reports improvements in the side lobe level (SLL) and half-power beam-width (HPBW), with values of -12.6 dB and 16.2°, respectively.

### Beam-steering based on phase shift array NA

**Fig. 13:**
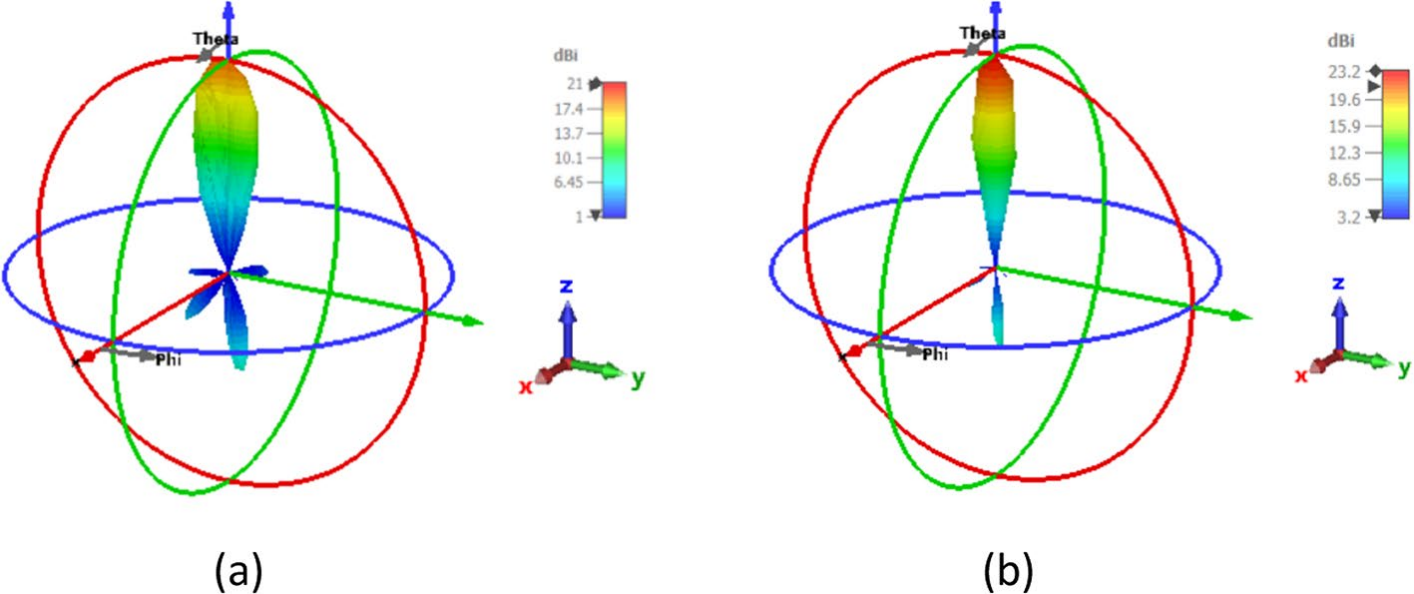
3-D radiation patterns of the array of SHHPNA with the square lens at 1550 nm of: (**a**) array of 4 ×1 at Δφ_X_ = 0°, (**b**) array of 3 ×3 at Δφ_X_, Δφ_Y_ = (0°, 0°).

This subsection describes the 2-D beam steerable antenna array operating at 1550 nm with a 4- and 9-element feed antenna array. Beam-steering using phase shift array NA is provided. An array of nano-antennas can be constructed to obtain the antenna with high gain, which is excellent for energy harvesting. As shown in Fig. 13, while the number of nano-antenna elements is increased, the gain is increased with values of 21 dBi and 23.2 dBi for the array of 4×1 and 3×3 of the SHHPNA with the square lens, respectively. This proves the effectiveness of the proposed nano-antenna for energy harvesting applications. Additionally, the SLLs and HPBWs have been enhanced, reaching -13 dB and 11.4° for the 4×1 NA array and -12.7 dB and 11° for the 3×3 NA array, correspondingly.

**Fig. 14:**
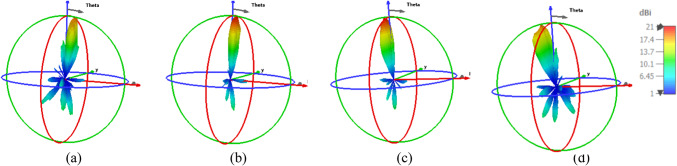
3-D radiation patterns of the array of 4 ×1 SHHPNA with the square lens at 1550 nm with: (**a**) Δφ_X_ = -90°, (b) Δφ_X_ = -45°, (**c**) Δφ_X_ = 45°, (**d**) Δφ_X_ = 90°.

By adjusting the relative phase between the antenna elements, beam-steering can be accomplished. Figure 14 demonstrates how the phase shift caused by a 4×1 array of SHHPNA with the square lens will alter the pattern’s orientation. The feeding phases of the NA array have been designed to vertically beam-steer the radiation pattern with a phase shift of (Δφ_X_ = −90°,-45°, +45°, +90°) at λ= 1550 nm. Gain, SLL, and HPBW values have also been shown in Table 3. The gain of 18.6 dBi is achieved by the 4x1 SHHPNA array with a lens that has a phase shift of +90°, as compared with 11.9 dBi for the conventional NA^37^.

**Table 3:** The radiation characteristic for 4×1 array of SHHPNA with lens.

Δφ_X_	Gain (dBi)	SLL (dB)	HPBW (°)
**-90°**	18.6	-8	12.4
**-45°**	20.7	-12	11.8
0°	**21**	**-13**	**11.4**
**45°**	20.7	-12.1	11.8
**90°**	18.6	-8	12.3

Significant values are in [bold].

**Fig. 15:**
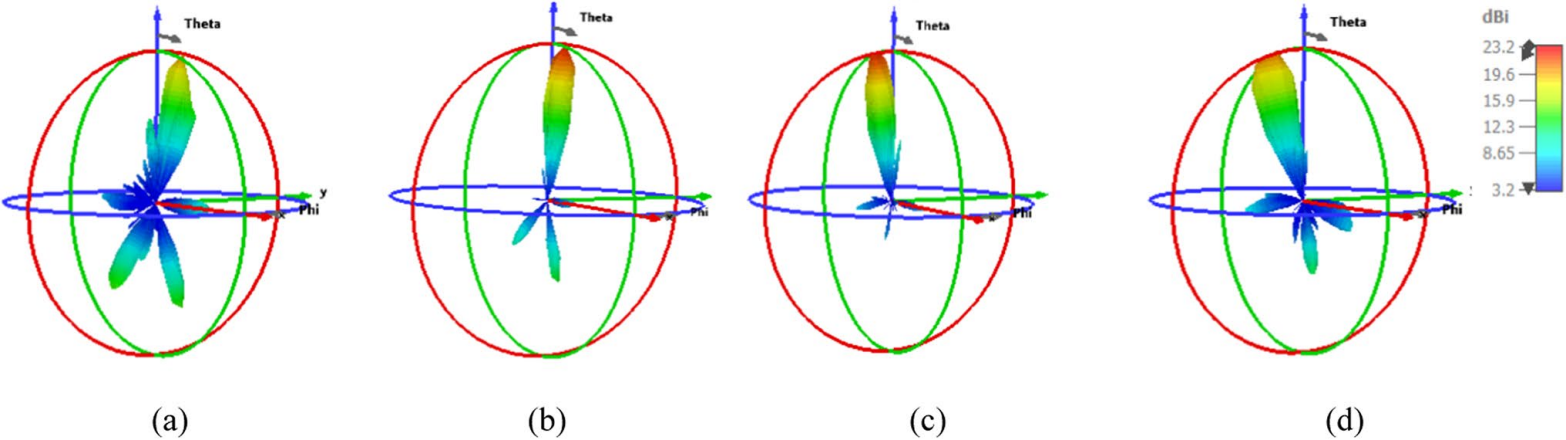
3-D radiation patterns of the array of 3 ×3 SHHPNA with the square lens at 1550 nm with: (**a**) Δφ_X_, Δφ_Y_ = (-90°, -90°), (**b**) Δφ_X_, Δφ_Y_ = (-45°, -45°), (**c**) Δφ_X_, Δφ_Y_ = (45°, 45°), (**d**) Δφ_X_, Δφ_Y_ = (90°, 90°).

Moreover, Fig. 15 depicts the far-field beam-steering radiation patterns of the 3× 3 array in various planes at a wavelength of 1550 nm, which are realized by optimizing the phases of light in the NAs with phase-shift in X and Y directions (Δφ_X_, Δφ_Y_) equal to (-90°, -90°), (-45°, -45°), (45°, 45°), and (90°, 90°). Table 4 displays the values for gain, SLL, and HPBW and demonstrates the controllability of the relative phase shift between the input optical signals in each feeding waveguide. For instance, each arm of the antenna array might incorporate very effective active phase shifters to accomplish this. The proposed nano-antenna in^37^ has a gain of 12.5 dBi for a 3x3 array, while the produced nano-antenna has a gain of 20.1 dBi for Δφ_X_ and Δφy = -90°. Moreover, the conventional NA’s 3x3 array produced a gain of 13.1 dBi, while the generated NA provided a gain of 20.7 dBi for Δφ_X_ and Δφy = 90°.

**Table 4:** The radiation characteristic for 3×3 array of SHHPNA with a lens.

Δφ_X_	Δφ_Y_	Gain (dBi)	SLL (dB)	HPBW (°)
**-90°**	-90°	20.1	-10.6	12.2
**-90°**	0°	21.8	-10.6	12.1
**0°**	-90°	21.4	-10.3	12.2
**-45°**	-45°	22.7	-12.2	11.1
**-0°**	-45°	22.7	-12	11.2
**-45°**	-0°	22.8	-12.2	11.1
0°	**0°**	**23.2**	**-12.7**	**11**
**45°**	0°	22.8	-12.3	11.1
**0°**	45°	22.7	-12	11.2
**45°**	45°	22.9	-12.3	11.1
**90°**	0°	21.9	-10.7	12.1
**0°**	90°	21.4	-10.2	12.2
**90°**	90°	20.7	-10.7	12.1

Significant values are in [bold].

**Fig. 16:**
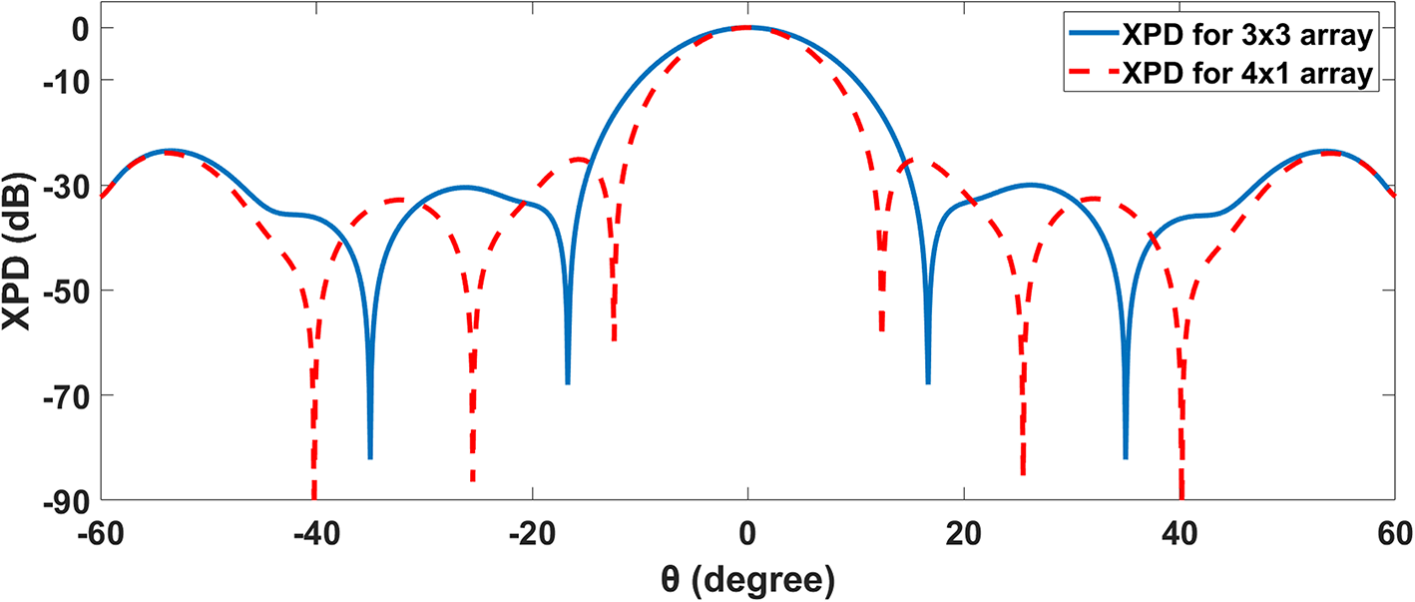
Cross-polar discrimination for 3x3, and 4x1 arrays of the proposed NA.

Cross-polarization discrimination (XPD) is a crucial variable in wireless communication systems, especially in antennas used to transmit and receive radio signals. It examines an antenna’s capability to reject or discriminate against signals polarized orthogonal to its intended polarization. XPD measures an antenna’s ability to isolate signals with different polarizations, minimizing interference and maximizing transmission performance. XPD is defined as the ratio of the co-polar component of the desired polarization to the orthogonal cross-polar component through the sector or beam-width angle. Figure 16 shows that the SHHPNA’s 3x3 array with the square lens has a greater XPD than the 4x1 array. A 3x3 array’s elements are organized in a grid, allowing for better control over the radiation pattern and polarization characteristics^66^. This configuration can more efficiently reduce the cross-polarized components than a linear 4x1 array. In addition, Mutual coupling between elements in a 3x3 array can be more efficiently regulated, resulting in better polarization separation. This can improve XPD by minimizing undesired cross-polarized signals. Therefore, a 3x3 array can provide more sophisticated beamforming capabilities, allowing for improved suppression of cross-polarized components. This is due to the larger number of elements and their geographical distribution.

## Conclusion

This paper describes a hybrid plasmonic nano-antenna with a gradient-index dielectric flat lens modelled with various materials to enhance and guide radiation in a certain direction. The design of hybrid plasmonic NA is introduced and analyzed using several horn shapes such as diamond, hexagonal, circular, square, and square. To analyze the radiation characteristics of the plasmonic NAs at the standard telecommunication wavelength of 1,550 nm, the commercial software Computer Simulation Technology-Microwave Studio (CST-MWS) is utilized. When compared to typical NA, the gain of the Square Horn-shaped Hybrid Plasmonic Nano-Antenna (SHHPNA) achieved 10.7 dBi, while the return loss reached 18.09 dB. Additionally, the performance of the antenna can be improved by employing two different types of dielectric flat lenses to reduce beam-width and increase directivity. The gain for SHHPNA with a circular lens and SHHPNA with a square lens is equal to 16.7, and 16.9, respectively. The main lobe for each lens is obviously more directed with Side Lobe Level (SLL) and Half Power Beam-Width (HPBW) of -13.1 dB and 16.5° for SHHPNA with a circular lens and -15.1 dB and 15.4° for SHHPNA with a square lens, respectively. Additionally, the array configuration was examined and produced for the single row array of 4×1 and the array of 3×3, and the gain achieved 23.2 dBi and 21 dBi, respectively.

## Methods

A 3D full-wave numerical simulation was run using CST software and the Uni-directional simulation setup with the boundary conditions set to open-add-space (modelling the radiation condition) in order to examine the performance of the complete structure. Two steps of the simulation were performed. In the initial stage, the inserted nano-antennas were examined using the Finite Elements technique. In step two, the lens was illuminated using the information from step one, and the array of nano-antenna elements was produced with a phase shift array to achieve beam-steering.

## Data Availability

The datasets used and/or analysed during the current study available from the corresponding author on reasonable request.
